# Burden of disease assessment for Germany and its regions – results from the BURDEN 2020 study

**DOI:** 10.1093/eurpub/ckac129.431

**Published:** 2022-10-25

**Authors:** M Porst, E von der Lippe, A Wengler, J Leddin, A Anton, A Rommel

**Affiliations:** Epidemiology and Health Monitoring, Robert Koch-Institute, Berlin, Germany

## Abstract

**Background:**

Epidemiological measures such as incidence, prevalence, or deaths are essential for monitoring population health. However, evaluating them in isolation cannot adequately compare and assess the relative importance of different diseases. Assessments of the burden of disease (BoD) are therefore of growing importance in supporting health policy decisions. Using disability-adjusted life years (DALY) as a summary measure of population health, BoD integrates morbidity and mortality in a transparent approach.

**Methods:**

Within BoD methodology, deviations in the health of the population from an ‘ideal’ health status is quantified in the unit of life years. DALY are the sum of years of life lost due to death (YLL) and years lived with disability (YLD). While YLL describe the gap between age at death and statistical life expectancy, the indicator YLD quantifies years lived with a disability or disease. Calculations were based on different primary and secondary data sources for Germany, especially cause-of-death statistics, epidemiological survey data, and statutory health insurance data.

**Results:**

In Germany, there were about 12 million DALY in 2017, the equivalent of 14,584 DALY per 100,000 population. Coronary heart disease contributes the most to the overall burden of disease, followed by lower back pain and lung cancer. In women, headache disorders and dementias account for more DALY as compared to men. Men have a higher burden of disease from lung cancer or alcohol use disorders. Pain disorders and alcohol use disorders lead the DALY rankings for both sexes in younger adulthood. The burden due to cardiovascular disease, COPD, and diabetes mellitus increases with age and also varies by region.

**Conclusions:**

The results suggest age- and gender-specific prevention as well as regional health care needs. BoD studies therefore provide comprehensive data for population health surveillance and can support health policy decisions.

**Key messages:**

• The importance of specific diseases as measured by DALY differs greatly by age and gender, highlighting the need for targeted prevention measures.

• Regional patterns emerge for cardiovascular disease, COPD, and depressive disorders, among others, which may indicate health care needs.

